# Temporal Patterns of Daily Behavioral and Psychological Symptoms of Dementia Throughout the Day

**DOI:** 10.1093/geroni/igab046.204

**Published:** 2021-12-17

**Authors:** Caitlin Connelly, Kyungmin Kim, Yin Liu, Steven Zarit

**Affiliations:** 1 University of Massachusetts Boston, Somerville, Massachusetts, United States; 2 Seoul National University, Seoul, Seoul-t'ukpyolsi, Republic of Korea; 3 Utah State University, Logan, Utah, United States; 4 Pennsylvania State University, Pittsburgh, Pennsylvania, United States

## Abstract

Behavioral and psychological symptoms of dementia (BPSD) are taxing for both the person with dementia (PWD) and their family caregivers. Yet, little is known about how BPSD fluctuates throughout the day (i.e., morning, daytime, evening, and night; e.g., sundowning) and how caregivers perceive BPSD at different times of the day. Using 8-day daily diary data from 173 family caregivers whose relatives were using Adult Day Services (ADS), this study investigated temporal patterns of BPSD and caregivers’ stress responses to BPSD throughout the day. Overall, the number of BPSD was highest in the evening, and caregivers’ stress reactivity to BPSD increased throughout the phases of the day (i.e., most stressful at night). However, caregivers showed lower reactivity to BPSD in the mornings and at night on days when the PWD used ADS. Our findings about fluctuations of (caregiver reactions to) BPSD throughout the day suggest target windows for just-in-time adaptive intervention.

